# Efficient Harvesting of *Nannochloropsis* Microalgae via Optimized Chitosan‐Mediated Flocculation

**DOI:** 10.1002/gch2.201800038

**Published:** 2018-09-21

**Authors:** Elvis T. Chua, Eladl Eltanahy, Heejae Jung, Manuel Uy, Skye R. Thomas‐Hall, Peer M. Schenk

**Affiliations:** ^1^ Algae Biotechnology Group School of Agriculture and Food Sciences The University of Queensland Brisbane Queensland 4072 Australia; ^2^ Algae Laboratory Botany Department Faculty of Science Mansoura University Mansoura 35516 Egypt; ^3^ The Johns Hopkins University Applied Physics Laboratory Laurel Maryland 20723 USA

**Keywords:** central composite design, chitosan, flocculation, microalgae harvesting, *Nannochloropsis*

## Abstract

Food‐grade rather than synthetic or chemical flocculants are needed for microalgae harvesting by settling, if used for food products. Chitosan is effective in harvesting freshwater microalgae, but it is expensive and typically not suitable for marine microalgae like *Nannochloropsis*. To minimize costs for food‐grade flocculation, a number of potentially important parameters are considered, including chitosan solubility and optimized chitosan‐mediated flocculation of *Nannochloropsis* sp. BR2 by a five‐factor central composite design experiment. Results show that an optical density (440 nm) of 2 (0.23 g dry weight L^−1^), initial pH of 6, final pH of 10, and 22 ppm chitosan with a viscosity of 1808 cP provide optimum flocculation efficiency, which is predicted to be in the range of 97.01% to 99.93%. These predictions are verified on 4.5 and 8 L *Nannochloropsis* sp. BR2 cultures.

## 1 Introduction

*Nannochloropsis* sp. and other microalgal species are prolific sources of nutraceuticals, but harvesting of microalgae is one of the factors that contribute to the high operating costs of microalgae farming. Currently, most industries utilize centrifugation to collect microalgal cells, which is capital intensive and consumes a high amount of energy. Flocculation has been considered as one of the most cost‐effective methods of microalgae harvesting.^1^ There are many flocculants that have been studied. The chemicals that are commonly used are inorganic salts such as ferric chloride (FeCl_3_) and aluminum sulfate (Al_2_(SO_4_)_3_) or organic polymers.^2^, ^3^, ^4^ On the other hand, natural biomaterials can be used as flocculants including biopolymers chitosan, which typically comes from the deacetylation of chitin from crab and shrimp shells^4^, ^5^ and poly(γ‐glutamic acid), an extracellular product from *Bacillus subtilis*.^6^ Modern techniques involve creating new materials such as nanoparticles,^4^ magnetic particles,^7^, ^8^ cationic polymers,^9^, ^10^ polymer composites,^11^ and even combinations of these materials.^12^ Inorganic salts have the disadvantage of contaminating the harvested biomass, which makes them not suitable for animal and human consumption. Nanoparticles and nanocomposites may be effective in flocculating microalgae in the laboratory scale. However, if these materials would be used at large scale microalgae production farms, currently, the cost of producing these materials are still too high leading to an expensive harvesting method.^13^ Thus, organic biopolymers are attractive flocculants to microalgae industries, especially if the biomass is to be sold as food‐grade.

Currently, the concern with the use of biopolymers is the cost. Numerous studies have shown that a very high amount of chitosan is needed to flocculate marine *Nannochloropsis* microalgal species, ranging from 30 to 200 ppm.^4^, ^5^ As chitosan is still expensive at $ 19–21 per kg (Qingdao Yunzhou Biochemistry Co Ltd), there is a need to further lower the optimum concentration for flocculation.

Initially, we used the inorganic salts Al_2_(SO_4_)_3_ and FeCl_3_ to flocculate *Nannochloropsis* sp. BR2. This experiment serves as the control since it is the commonly used method being used in large‐scale algae farms. Furthermore, we optimized the method for flocculating *Nannochloropsis* sp. BR2 using chitosan via a five‐factor central composite design (CCD) experiment that introduced strategic pH changes. The results obtained from the CCD experiments were tested on a larger scale, yielding flocculation at lower concentrations.

## 2 Results and Discussion

### 2.1 *Nannochloropsis* sp. BR2 Flocculation Using Al_2_(SO_4_)_3_ and FeCl_3_

**Figure 1:**
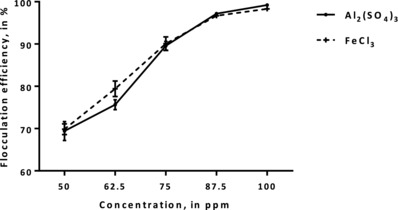
FE of *Nannochloropsis* sp. BR2 using different concentrations of Al_2_(SO_4_)_3_ and FeCl_3_.

Aluminum sulfate and ferric chloride were effective in flocculating *Nannochloropsis* sp. BR2 cells. **Figure** **1** shows that the effective concentration for Al_2_(SO_4_)_3_ and FeCl_3_ was 87.5 ppm, resulting in 95.2% (±1.1) and 95.6% (±1.2) flocculation efficiency (FE) values, respectively.

Further increasing the concentration of both inorganic flocculants increased the FE by only 2–2.3%. Inorganic salts are able to flocculate microalgal cells by charge neutralization.^2^, ^14^ Increasing the concentration of inorganic salts also increases the amount of cations capable of neutralizing the negative microalgal cell charge by electrostatic interactions.

### 2.2 *Nannochloropsis* sp. BR2 Flocculation Using Chitosan Without pH Change

**Figure 2:**
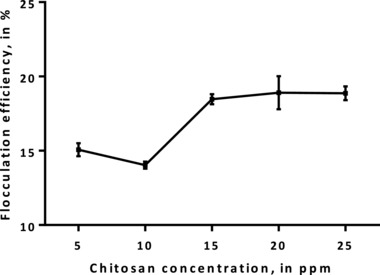
FE of *Nannochloropsis* sp. BR2 using different chitosan concentrations without pH change.

To make chitosan cost‐effective compared to the inorganic salts, lower concentrations should be used. Thus, concentrations ranging from 5 to 25 ppm were tested as shown in **Figure** **2**. In the chitosan concentration range studied, the FE values did not reach 20%, showing that chitosan is not effective in this range. Lama et al. even showed that chitosan is not able to induce the formation of *Nannochloropsis* sp. flocs.^15^ As the chitosan was dissolved in acetic acid, the pH of the culture decreased to 6–7. At this pH, chitosan is positively charged because of the peripheral amino groups.^16^ As such, flocs should be expected to form since the negatively charged microalgal cells will be neutralized by the positively charged chitosan polymers. However, since the microalgae were grown in brackish conditions, most of the positive charges of the chitosan molecules are shielded due to the ionic strength. Consequently, the adsorption between the microalgal cells and chitosan molecules are reduced.^17^, ^18^

### 2.3 Central composite design

CCD was applied to optimize five variables for the flocculation of *Nannochloropsis* sp. BR2. The experimental results of the 28‐run design can be found in **Table** **1**. Multiple regression analyses were applied on the acquired data. A multiple correlation coefficient (*R* ^2^) of 0.96 indicated that the model was suitable for representing the relationship of factors and predicting the response values. Aside from this, results showed that out of the five factors, only the initial pH of the culture did not significantly contribute to the model. This observation is most likely because chitosan is protonated at pH 6–7 and the addition of the chitosan solution already decreases the initial pH to around 6.8.^17^ The other four factors significantly affected the model. OD and the chitosan concentration significantly affected FE because higher OD and chitosan concentrations mean that more material is available for the formation of flocs. The final pH of the mixture also affected the FE because chitosan becomes insoluble in water at pH higher than 7.5.^19^ This property causes chitosan precipitates to form that entrap the microalgal cells in a net‐like structure.^20^ Finally, chitosan viscosity significantly affected the FE because increasing chitosan viscosity is caused by increasing the extent of deacetylation;^21^, ^22^ consequently, increasing the positively charged surface area for chitosan‐microalgae interaction when the pH is lowered to 6.8.

**Table 1:** Factor table for the central composite design for five variables with the experimental and predicted responses

Run	OD	Initial pH	Final pH	Chitosan concentration [ppm]	Chitosan viscosity [cP]	Observed FE [%]	Predicted FE [%]
1	1.05	7	9	15	1600	76.2	79.3
2	1.05	8	9	15	1600	71.4	81.1
3	0.1	8	10	5	17	2.0	2.2
4	1.05	7	9	5	1600	55.5	54.0
5	2	7	9	15	1600	96.6	95.2
6	2	6	8	25	17	68.2	67.9
7	1.05	7	8	15	1600	16.9	40.4
8	1.05	6	9	15	1600	94.2	89.9
9	0.1	8	8	5	3600	12.0	9.5
10	0.1	6	10	5	3600	71.0	73.6
11	0.1	6	8	5	17	7.0	6.4
12	2	6	10	5	17	83.1	87.4
13	0.1	6	10	25	17	56.0	61.9
14	1.05	7	9	15	1600	78.4	79.3
15	2	8	10	25	17	94.0	94.9
16	0.1	8	10	25	3600	97.0	97.0
17	1.05	7	9	25	1600	80.5	80.5
18	2	8	8	25	3600	51.0	46.0
19	2	8	8	5	17	33.2	29.7
20	1.05	7	9	15	17	48.4	40.2
21	0.1	7	9	15	1600	47.0	54.5
22	0.1	6	8	25	3600	8.0	7.0
23	2	6	10	25	3600	98.7	98.9
24	1.05	7	10	15	1600	97.6	92.0
25	0.1	8	8	25	17	5.0	3.9
26	2	6	8	5	3600	63.7	62.1
27	1.05	7	9	15	3600	68.4	74.0
28	2	8	10	5	3600	92.7	93.2

**Figure 3:**
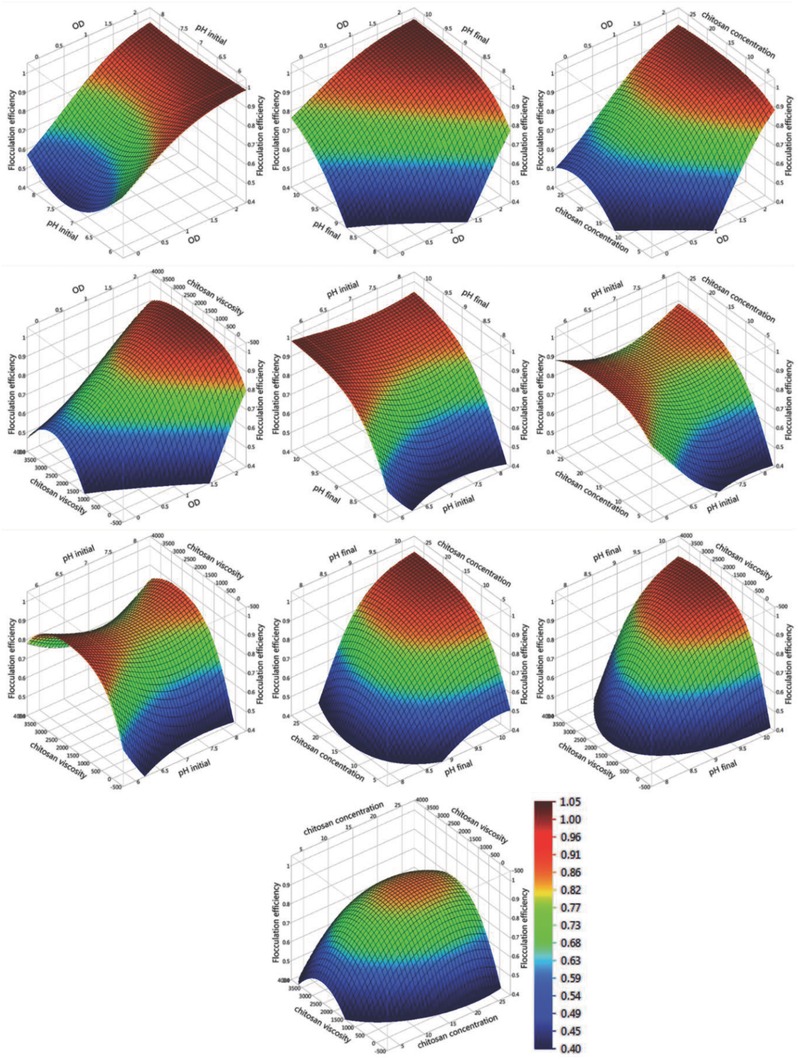
Response surface plots of FE for the five‐variable CCD flocculation experiments.

By using the generated model, the best setting that provided the highest FE was an OD of 2, initial pH of 6, final pH of 10, 22 ppm chitosan, and 1808 cP chitosan viscosity (replaced with 1600 cP chitosan due to availability). These optimum parameters are evident in the response surface plots (**Figure** **3**) as they provide the maximum FE values. Verification tests showed that the FE (97.9% ± 1) is within the predicted FE range of 97.01–99.93%. The optimum chitosan concentration is lower than other studies as shown in **Table** **2**.

**Table 2:** *Nannochloropsis* sp. chitosan flocculation dosage comparison with other references

Species	Concentration [ppm]	pH	FE [%]	Reference
*Nannochloropsis* sp.	60a)	9	97	5
	100	9	90	
*Nannochloropsis gaditana*	30	9.9	85	23
*Nannochloropsis* sp.	30	n.a.	95	24
*Nannochloropsis* sp.	150	6.5 → 8	95	25
*N. gaditana*	150		95	
*Nannochloropsis oculata*	No flocculation observed	n.a.	n.a.	15
*Nannochloropsis* sp. BR2	22	6 → 10	97.9	This study

^a)^ Using nanochitosan.

### 2.4 Testing Optimized Parameters at Larger Scale

**Figure 4:**
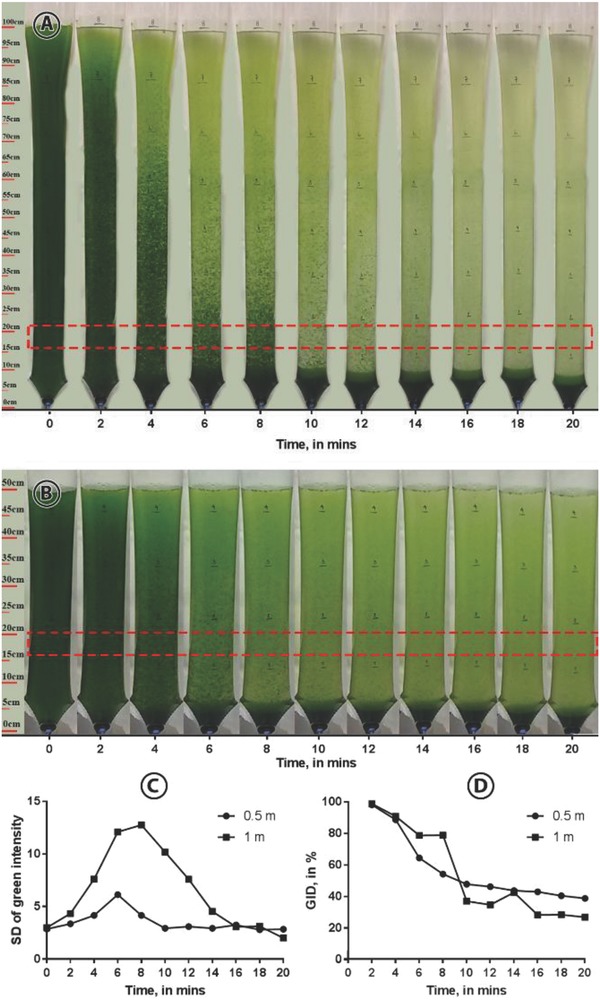
Images of A) the 1 m and B) 0.5 m culture flocculated using the optimized parameters. Each image was taken at 2 min intervals. The rectangles indicate the areas where the green intensities were measured. C) Standard deviation (SD) of green intensity of the images taken. D) Percent difference in the green intensity of the images taken.

The optimized parameters were tested on larger *Nannochloropsis* sp. BR2 cultures: 4.5 L (0.5 m depth) and 8 L (1 m depth). These volumes were selected to test the effect of the culture depth on FE. **Figure** **4**A,B shows that the larger flocs start to form and settle during 2–10 and 2–16 min for the 0.5 and 1 m cultures, and these observations were confirmed by the standard deviation of the green intensity (Figure 4C). Finally, the green intensity difference shows that the final green intensity left in the 1 m culture was lower than that of the 0.5 m culture (Figure 4D). This observation is most likely due to the higher pressure present in the 1 m culture; thus, forcing more of the flocs down to the bottom of the bioreactor. In fact, after 20 min settling, FE values were at 93.4% and 97.2% for the 0.5 and 1 m cultures, respectively. Preliminary tests have shown that repeat cultivation in recycled water from these cultures did not affect growth compared to cultures harvested by centrifugation.

The current study demonstrates that chitosan instead of chemical flocculants was a viable option for the harvesting of *Nannochloropsis* algae. Although only 22 ppt chitosan was needed to harvest >97% (compared to 87.5 ppm of Al_2_(SO_4_)_3_ or FeCl_3_), the costs are still estimated to be 9–34 times higher ($ 0.15 per kg Al_2_(SO_4_)_3_ (Zibo City Linzi Yixiang Chemical Co Ltd); $ 0.53 per kg FeCl_3_ (Changsha Choice Chemicals Ltd)). Nevertheless, the option to use chitosan would still be preferred if high‐value products are derived from *Nannochloropsis* such as eicosapentaenoic acid^26^ or carotenoids, which may also be produced in a multiproduct biorefinery setting^27^ or with a closed nutrient loop.^28^ Furthermore, chitosan is also available from fungi, including edible mushrooms,^29^ and its use for *Nannochloropsis* flocculation may present a desirable choice for the production of high‐value, algae‐derived nutraceuticals for vegans.

## 3 Conclusions

Use of chitosan for *Nannochloropsis* harvesting is practicable at concentrations as low as 22 ppm when using optimized parameters including pH changes. To obtain high FE only about a fourth of its inorganic counterparts are needed. Even though chitosan still costs significantly more than inorganic flocculants, microalgae harvesting with chitosan could be a suitable method to produce food‐grade biomass for human food supplements and a vegan option is available if sourced from fungi.

## 4 Experimental Section

*Algal Strain and Cultivation Conditions: Nannochloropsis* sp. BR2 was obtained from the University of Queensland Algae Biotechnology culture collection.^30^ The isolate was scaled up in a 20 L polyethylene bag culture. The microalgae were grown with a salinity of 20 ppt using Ocean Nature Sea Salt enriched with F/2 medium at a pH of 8.5. Samples for the flocculation experiments were collected at the end of the exponential phase with an optical density (OD) at 440 nm of 2.5 (or 0.28 g dry weight L^−1^) measured at 440 nm.

**Figure 5:**
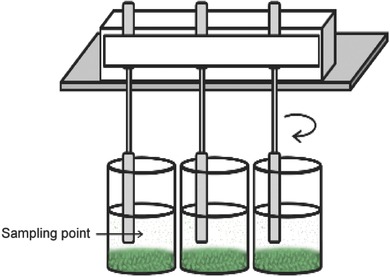
A schematic representation of the improvised multistirrer for the flocculation experiments. The curved arrow indicates the movement of the stirrer.

*Preparation of Flocculants and Use of Multistirrer*: Stock solutions (5000 ppm) of Al_2_(SO_4_)_3_ and FeCl_3_ salts were prepared in deionized water. On the other hand, different concentrations (1000, 2000, 3000, 4000, and 5000 ppm) of chitosan stock solutions were freshly prepared in 0.1 m acetic acid. All chemicals except for the chitosan were obtained from Sigma. The chitosan samples with various viscosities were provided as test samples by Austanz Chitin Pty Ltd, Queensland, Australia. A custom‐made multistirrer was used for the flocculation experiment. It consists of several metal spatulas attached to a support that is placed on top of an orbital shaker as shown in **Figure** **5**. The mixing speed was controlled using the orbital shaker and for the flocculation experiments, the speed was set to 100 rpm.

*Flocculation Experiments*: The pH of the culture was adjusted to 8.0 and then 100 mL samples were placed in 250 mL polycarbonate jars. A certain amount of flocculants was added to each jar to obtain the final desired concentration. Samples were then mixed for 15 min using the stirrer described before. All samples were set aside for 30 min to allow the clumps to settle. One milliliter of sample was collected at half the height of the 100 mL sample as shown in Figure 5 and the OD was measured at 440 nm. The flocculation efficiency was computed as

(1) $$\mathrm{Flocculation}\;\mathrm{efficiency}\;(\mathrm{FE},\;\mathrm{in}\;\%)\;=\left(1-\frac{{\mathrm{OD}}_{\mathrm{sample}}}{{\mathrm{OD}}_{\mathrm{control}}}\right)\;\times \;\mathrm{100}$$

*Central Composite Design*: Preliminary results showed that chitosan cannot be used to harvest *Nannochloropsis* sp. BR2 without changing the pH of the sample. This observation led to the need of optimizing two more variables, the initial pH and the final pH of the sample, since the charge of chitosan changes as the pH changes. A modification of the classical flocculation method was developed. After the addition of chitosan, the pH of each sample was adjusted to the desired initial pH level by the addition of either 0.1 m HCl or NaOH. Samples were mixed for 15 min. Stirring was stopped and pH was adjusted to the desired final pH. Samples were then mixed again for 15 min. In addition, two more factors, culture OD and chitosan viscosity, were added to aid in understanding the flocculation mechanism.

CCD was used to study and optimize five variables, namely, 1) culture OD, 2) initial pH of the culture, 3) final pH of the culture, 4) chitosan concentration, and 5) chitosan viscosity. CCD requires each variable to have three levels as shown in **Table** **3**. JMP version 13.0.0 was used to generate the factor table and to perform the necessary statistical analyses.^31^ The FE values were subjected to logit transformation to limit the value from 0 to 1. By using the optimized parameters as predicted by the derived model, verification tests were performed in triplicates.

**Table 3:** Factors and levels of the central composite design

Factor	Level		
	−1	0	+1
Culture OD	0.1	1.05	2
Initial pH	6	7	8
Final pH	8	9	10
Chitosan concentration [ppm]	5	15	25
Chitosan viscosity [cP]	17	1600	3600

*Testing Optimized Parameters at a Larger Scale*: Two polyethylene bags with 4.5 and 8 L *Nannochloropsis* sp. BR2 culture were prepared. The depths of the cultures were 0.5 and 1 m, respectively. After the addition of chitosan, each culture was mixed for 10 min using an air blower. The pH was then adjusted using 0.1 m HCl or NaOH to the optimized pH values. Finally, the culture was mixed again for 10 min. The bubbling was stopped to allow the flocs to settle. Images were taken every 2 min to monitor the settling of the flocs. A portion of the image, which is at the 15–20 cm mark was cut and subjected to image analysis using ImageJ. Each image was split into its red‐green‐blue (RGB) channels, and only the green channel was used for the analysis. The mean intensity of each image was obtained and compared with the image taken at 0 min. The equation below was used to serve as a quantitative measure of how the flocs settled.

(2) $$\mathrm{Green}\;\mathrm{intensity}\;\mathrm{difference}\;(\mathrm{GID},\;\mathrm{in}\;\%)\;=\left(1-\frac{{\mathrm{MGI}}_{t}}{{\mathrm{MGI}}_{0}}\right)\times \mathrm{100}$$

where MGI*_t_* is the mean green intensity at *t* min and MGI_0_ is the mean green intensity at 0 min. After 20 min, the dense culture that settled was drained and the supernatant was collected. The FE values were computed as described above.

*Statistical Analyses*: Data and error bars in figures are presented as the mean and standard error of triplicates.

## Conflict of Interest

The authors declare no conflict of interest.

## Supporting information

Supplementary
