# Supplementary material for: Efficient Harvesting of Nannochloropsis Microalgae via Optimized Chitosan‐Mediated Flocculation
Source: Glob Chall. 2018 Sep 21;3(1):1800038. doi: 10.1002/gch2.201800038 (PMC6383959; doi:10.1002/gch2.201800038)
Supplement: Supplementary file 1 — Supplementary [file GCH2-3-1800038-s001.pdf]

Copyright WILEY-VCH Verlag GmbH & Co. KGaA, 69469 Weinheim, Germany, 2018.

Global Challenges
Open Access

Supporting Information

for *Global Challenges,* DOI: 10.1002/gch2.201800038

Efficient Harvesting of *Nannochloropsis* Microalgae via Optimized Chitosan-Mediated Flocculation

*Elvis T. Chua, Eladl Eltanahy, Heejae Jung, Manuel Uy, Skye R. Thomas-Hall, and Peer M. Schenk**

Supplementary data

Table S1. Statistical results of the regression model for FE.

Source	Degrees of freedom	Sum of squares	Mean square	F value	Prob > F
Model	20	117.17	5.86	8.75	0.0034 **
A-OD	1	1	33.19	49.58	0.0002 **
B-initial pH	1	1	1.89	2.83	0.1367
C-final pH	1	1	39.36	58.81	0.0001 **
D-chitosan concentration	1	1	6.61	9.87	0.0164 *
E-chitosan viscosity	1	1	9.37	13.99	0.0073 **
AB	1	1	0.08	0.12	0.7404
AC	1	1	0.00	0.00	0.9562
BC	1	1	0.00	0.01	0.9364
AD	1	1	0.26	0.40	0.5494
BD	1	1	1.55	2.31	0.1722
CD	1	1	5.63	8.41	0.0230 *
AE	1	1	2.38	3.55	0.1014
BE	1	1	2.02	3.02	0.1256
CE	1	1	4.62	6.90	0.0341 *
DE	1	1	0.57	0.85	0.3878
A^2	1	1	0.14	0.21	0.6626
B^2	1	1	0.55	0.83	0.3930
C^2	1	1	0.25	0.37	0.5642
D^2	1	1	0.76	1.13	0.3227
E^2	1	1	2.99	4.47	0.0724
Lack Of Fit	6	4.68	0.78	100.03	0.0764
Pure Error	1	0.01	0.01		
Total Error	7	4.69			

*$p < 0.05$

**$p < 0.01$
